# Attitudes and burden in relatives of patients with schizophrenia in a middle income country

**DOI:** 10.1186/1471-2296-12-101

**Published:** 2011-09-26

**Authors:** Alejandra Caqueo-Urízar, José Gutiérrez-Maldonado, Marta Ferrer-García, Claudia Peñaloza-Salazar, David Richards-Araya, Alejandro Cuadra-Peralta

**Affiliations:** 1 Departamento de Filosofía y Psicología, Universidad de Tarapacá, 18 de Septiembre # 2222, Arica, Chile; 2 Departamento de Personalidad, Evaluación y Tratamiento Psicológico, Facultad de Psicología, Universidad de Barcelona, Paseo Valle de Hebrón, 171, 08035 Barcelona, Spain; 3 Equipo de Psiquiatría y Salud Mental Ambulatoria, ESSMA SUR, Vicuña Mackenna # 630, Arica, Chile

**Keywords:** Attitude, burden, relatives, schizophrenia

## Abstract

**Background:**

Most studies of family attitudes and burden have been conducted in developed countries. Thus it is important to test the generalizability of this research in other contexts where social conditions and extended family involvement may be different. The aim of this study was to assess the relationship between the attitudes of caregivers and the burden they experience in such a context, namely Arica, a town located in the northernmost region of Chile, close to the border with Peru and Bolivia.

**Methods:**

We assessed attitudes towards schizophrenia (including affective, cognitive and behavioural components) and burden (including subjective distress, rejection and competence) in 41 main caregivers of patients with schizophrenia, all of whom were users of Public Mental Health Services in Arica.

**Results:**

Attitude measures differed significantly according to socio-demographic variables, with parents (mainly mothers) exhibiting a more negative attitude towards the environment than the rest of the family (t = 4.04; p = 0.000).This was also the case for caregivers with a low educational level (t = 3.27; p < 0.003), for the oldest caregivers (r = 0.546; p = 0.000) and for those who had spent more time with the patient (r = 0.377; p = 0.015). Although attitudes had significant association with burden, their explanatory power was modest (R^2 ^= .104, *F *= 4,55; *p *= .039).

**Conclusions:**

Similar to finding developed countries, the current study revealed a positive and significant relationship between the attitudes of caregivers and their burden. These findings emphasize the need to support the families of patients with schizophrenia in this social context.

## Background

In Chile, with a population of 16 million people, the prevalence of schizophrenia ranges between 1.4-4.6 persons for every hundred thousand people, with the annual incidence rate being 12 new cases for every hundred thousand inhabitants [1]. The prevalence of this disorder is higher in the Arica-Parinacota region and in the capital, Santiago [2]. Further, a National Health Survey carried out in 2004 recorded 917,939 Chileans (5.7% of the population) as being moderately/severely disabled, with 68.71% of these being taken care of by relatives [1].

At the beginning of the 1990s, and together with the return to democracy in Chile, mental health reforms began to be implemented and the country witnessed a progressive increase in government investment in the area, the development of psychiatric and mental health service networks, and the creation of user groups. Although these changes led to the emergence of a community-centred model of care, reform has been partial and unequal across the country. It was partial because these developments did not translate into adequate social services and benefits, despite the meaningful advances made in terms of healthcare. And it was unequal since large differences remain between different regions of Chile, with a greater need in the regions [3]. In this regard, research conducted in the country's second largest city reported lower levels of caregiver burden, in contrast to the intense burden found among caregivers in the extreme north of Chile. This may be due to the fact that the mental health system in the north of the country has minimal resources for patient and family care [4,5].

At all events the closing of mental institutions has enhanced the study of mental disorders from a family perspective. That is, research has focused on emotional and affective states not only in the patient's psycho-social environment but also in his/her home [6,7]. It is in this context that burden needs to be understood. Dillehay and Sandys [8] defined *caregiver burden *as a psychological state that appears as a combination of physical and emotional work, social pressure, and financial restrictions which are consequences of taking care of a patient. This construct has been understood as any potentially verifiable and observable disturbance in the life of the caregiver that is caused by the patient's disease and which appears as a result of supervision and stimulation tasks [9,10]. The psychological distress perceived by caregivers is related to multiple factors, such as the caregiver's personality, the patient's psychological symptoms, and the availability of social support and financial resources [5,11-13].

From this perspective, the caregiver is conceptualized as an individual belonging to the patient's informal support system (a relative or friend) who takes responsibility for the main care tasks and who is perceived within the family as the person in charge of the patient, without being financially rewarded for this work [14]. This person is usually referred to as the 'main caregiver', the one who dedicates most of his/her time (number of hours per day) to caring for the patient [15]. With patients who have a severe mental disorder the role of main caregiver has usually been taken by relatives. As such patients cannot perform many of their personal and social duties caregivers have to stand in for them. Furthermore, they need continuous assistance with daily life, as well as frequent help in containing disturbed behaviours related to their disorder [4,16-18].

One study with a Latin American sample [4] found that relatives of patients with schizophrenia showed high levels of burden as a result of the care task. The financial problems, the restriction of spare time, and the patient's future, were of considerable concern to caregivers. In this context it is helpful to distinguish between *objective *and *subjective burden*. The former includes any disturbance in the family environment due to the patient's disorder and which is potentially verifiable and observable. Operatively, objective burden refers to disturbances in work, social life, spare time, housework, finances and interactions both within and outside the family [19,20]. By contrast, subjective burden refers to the feeling of being subjected to a heavy and oppressive duty, and to the subjective reaction of caregivers with respect to the tasks they have to perform [21].

Another important aspect relates to social networks. Research has demonstrated that caregivers are more likely to face restrictions in their social activities, thereby reducing their own social networks [22]; they may therefore remain isolated in their homes with few social contacts. Stigma about mental illness is also still frequent in many families and can contribute to social isolation [23,24]. Indeed, guilt and shame seem to be related to discrimination as a form of social avoidance [25]. Three large studies reported that between a fifth and a third of family members showed concern and maintained distant relationships with the rest of the family and friends because of the mentally disturbed relative [26-28]. By contrast, research has found that multiple roles among caregivers can reduce stress by expanding their available resources [29]. For example, working outside the home seems to be a good shield against burden as it can provide access to social networks in the form of interpersonal interactions and social support [30]. It has been shown that caregivers of people with a mental disability who spend more hours working outside the home experience significantly less stress in comparison with caregivers who do not [29].

In summary, these studies suggest that burden levels a) can change over time, b) are influenced by the nature of the patient's symptoms, and c) will probably not decrease without specific interventions [31].

As regards attitudes, these have been defined as "predispositions to associate certain kinds of stimuli with certain kinds of responses" [32]. Such responses are classified as *affective *(assessing feelings as pleasant or unpleasant), *cognitive *(concerning beliefs, opinions, and ideas about the attitude object) and *behavioural *(concerning behavioural intentions or action predispositions). This leads to a *three-component attitude model*, in which attitudes constitute a hypothetical construct mediating between the observable preceding stimulus and the subsequent behaviour. The present research is based on this multi-component model.

With regard to the attitudes of relatives of patients with schizophrenia it should be noted that the social stigma attached to mental disorders contributes to feelings of frustration and anger. Families are forced to acknowledge the stark reality of having a member with schizophrenia and to mourn the loss of unfulfilled expectations. Moreover, as a result of the chronic stress associated with the task of caring, the family may experience a series of marital conflicts between parents or differences in relation to the other siblings [33]. Thus, it is common for families to have emotional responses such as anxiety, fear, guilt, stigma, frustration, anger, sadness and so on. Furthermore, these family conflicts contribute to the stress experienced by its members [34-36]. Families may also develop other behavioural responses, such as adapting to the situation as if it were normal, resorting to prayer, finding meaning in the patient's communications, ignoring the patient's behaviour, or taking on additional responsibilities, and this can sometimes lead to overprotection.

Research on relatives' attitudes towards patients with schizophrenia began in 1959, when Brown and colleagues studied the effect of a patient returning home after hospitalization [37]. They found that patients who returned to live with their parents or partners had a higher number of relapses than did those returning to another type of living environment. Subsequent research thus began to evaluate the relationship between caregiver attitudes, patients' social functioning, caregivers' quality of life, caregiver burden, and the effectiveness of family intervention [38-40].

The assessment of patients who returned home after hospitalization led to two main findings: a) close emotional ties are not always beneficial for patients; and b) the continuing close contact between the patient and the family may be associated with relapse. Interest in the notion of close affective ties saw research being conducted into expressed emotion (EE) in the family. This showed that in families of patients with schizophrenia that are characterized by high EE, the rate of relapse is higher (48%) than in those with a low EE (6%) [41]. This research argued that although the aetiology of schizophrenia remains unknown, it is possible to talk about factors that precipitate relapse and which are linked to pharmacological and social influences (such as the family's EE). Initial work in this area reported that high levels of EE in the family were repeatedly found to predict rates of relapse in patients with schizophrenia nine months after discharge [42]. A stressful family environment is an important risk factor, and the risk of relapse is about four times higher in patients returning to a family environment with high levels of EE. By contrast, low EE caregivers show greater acceptance towards their relative with schizophrenia, and this is related to lower levels of distress in both patient and caregivers [43].

Given the above it appears that the attitudes underlying the construct of EE, namely the criticism, hostility and emotional over-involvement (EOI) shown by relatives, have a significant impact on the course of the disorder, as well as on caregiver burden. However, research suggests that these attitudes may differ across ethno-cultural groups. Several studies found that Mexican Americans usually show lower rates of EE than do European Americans [44-47]. This research emphasizes that Mexican American caregivers are less critical, less hostile and show more warmth towards their relatives with schizophrenia than do Anglo-American caregivers. Other studies have suggested that ethno-cultural differences also affect the nature of the association between EE and relapse in patients with schizophrenia and the health status of caregivers [48,49]. Thus, while criticism is considered the main contributor to patient relapse and caregiver burden in Anglo-American samples, EOI seems to be the main predictor of worse health outcomes and burden in Mexican American caregivers [48]. Latin Americans generally show high levels of engagement with their ill relatives and usually understand and empathize with their problems. However, Breitborde and colleagues [50] suggested that only moderate levels of EOI were associated with better outcomes, both in patients and caregivers. When EOI is too high or too low, patient relapse and caregiver burden rates increase again. All these studies highlight the need to take into account ethno-cultural aspects when studying caregivers' experiences.

Another study reviewing the relationship between family attitudes and the social functioning of patients with schizophrenia [51] found a significant correlation between an empathic attitude and the patient's social and occupational functioning. Caregivers who show more tolerant, non-intrusive and supportive attitudes towards patients help them to achieve better social functioning. Thus, more empathic caregivers can adjust their behaviour according to the patient's emotional state and needs, which protects them from extreme reactions that could eventually trigger a relapse. Furthermore, these empathic attitudes promote the generation of constructive ideas and facilitate the resolution of problems, which can help identify social opportunities. The authors of this study suggest that improving caregiver attitudes must be a part of family intervention programmes. Recently, Gutiérrez-Maldonado and colleagues [52] assessed the efficacy of a family psycho-educational programme designed to change attitudes and health perceptions in relatives of patients with schizophrenia. The results showed that the programme was effective in modifying caregiver attitudes, although it had no effect on their health perceptions. The programme also increased levels of satisfaction regarding the course of the patient's disorder [53].

Based on these outcomes, greater emphasis is now placed on psycho-educational interventions, since they not only provide training, emotional reinforcement, information and social support, but also improve relatives' attitudes towards schizophrenia, which in turn impacts on the caregiver's quality of life [51,54-56]. Dixon and colleagues [57] found that a family intervention programme led to significantly greater family, community and health service empowerment, and also reduced displeasure and concern about the family member with a mental disorder. Group-based interventions enable families to share experiences with others in similar situations, which can provide comfort and facilitate the expression of feelings about the disorder, thereby improving coping skills. It has been suggested that such intervention groups also increase the motivation of family members involved. Finally, it should be noted that psycho-educational programmes do not only provide information, but also reinforce the idea of respect for families and encourage them to consider themselves as co-therapists in the process. In this way, the therapeutic team and the family can develop a less polarized and less stressful relationship, and even more reluctant family members become more willing to cooperate, thereby reducing the burden on both parties [58].

Multifamily interventions have also considered the attitudes of caregivers. Weissman and colleagues [6] found a significant relationship between caregiver emotions and supportive behaviours toward the patient, and when caregivers showed a more sympathetic attitude towards patients they also made a greater effort to help them cope with their condition. These authors also conducted a qualitative analysis of the beliefs, values and behaviours of family members in order to gain a better understanding of their low EE. They found three main categories of attributes: a) the majority of caregivers assumed that the patient had a legitimate mental disorder; b) external environmental stressors were regarded as the cause of the condition or its worsening; and c) many caregivers implicated God in their attributions about the disorder and indicated that religion had become a source of hope about the situation. The authors concluded that the promotion of positive and favourable emotions among caregivers could be a key step in terms of establishing a low EE environment. At all events, the limited expression of negative affect reported in this sample should be seen as reflecting the Latin American cultural tendency towards social desirability, which together with a suppression of negative affect may be a factor that contributes to favourable outcomes in the patient [6].

Despite all the above findings there is still a gap between outcomes and service implementation. Moreover, this difference is more marked in developing countries that are characterized by different cultural values and a scarcity of available resources [59,60]. Given the importance of this issue, which is considered a priority area by policy makers (including in Chile), and the lack of research in different cultural contexts, the aim of the present study was to assess attitudes and burden among caregivers of patients with schizophrenia, in this case, patients who were users of public mental health services in Arica, Chile. The first step involved developing the *Attitudes Towards Schizophrenia Questionnaire for Relatives*, which covers three attitudes components: emotional, cognitive and behavioural. We then took into account that previous studies have found that the schizophrenic relative's symptoms affect both caregiver attitudes and perceived burden [61,62]. Furthermore, several authors consider that contextual variables, such as demographic characteristics of caregivers and relatives with schizophrenia, may modulate the effect of patients' symptoms on the emotional state of caregivers [6,63]. In light of this, a second aim was to assess differences in the attitudes of caregivers according to the social and demographic characteristics of both caregivers and patients. Specifically, with regard to patient characteristics, we expected that symptom severity, the number of hospitalizations and the receipt of a disability pension would affect caregiver attitudes. As regards caregiver characteristics, we expected that attitudes would be influenced by working outside the home (employment). Finally, a third aim of the study was to assess the relationship between caregiver attitudes and the burden they experience. We expected that more negative attitudes would lead to greater perceived burden.

## Method

### Participants

The sample was drawn from the population of relatives and caregivers of patients with schizophrenia who were attending public mental health centres in the city of Arica, Chile. Forty-five relatives and caregivers of patients with schizophrenia were initially recruited, but four dropped out. Tables 1 and 2 show the socio-demographic characteristics of patients and caregivers.

**Table 1:** Socio-demographic characteristics of caregivers

Caregivers	N = 41
**Age**	54.2 years (± 15.0)

**Years living with the patient**	25.9 years (± 13.0)

**Gender**	

Male	31

Female	10

**Marital Status**	

Single	6

Married	24

Divorced	6

Widowed	5

**Educational Level**	

Elementary	23

Secondary	10

Technical Studies	6

Professional Studies	2

**Employed**	

Yes	18

No	23

**Participation in psycho-social Programme**	

Yes	21

No	24

**Kinship with the patient**	

Father	6

Mother	26

Spouse/partner	4

Child	1

Brother	2

Sister	2

Other	5

**Table 2:** Clinical and Socio-demographic characteristics of patients

Patients	N = 41
**Age**	33.2 years (± 8.4)

**Average age at onset**	23.2 years (± 8.6)

**Number of hospitalizations in the last three month**	0.8 (± 1.3)

**Total number of hospitalizations**	3.4 (± 3.9)

**Gender**	

Male	26

Female	15

**Diagnosis**	

Paranoid Schizophrenia	26

Residual Schizophrenia	6

Catatonic Schizophrenia	3

Hebephrenic Schizophrenia	3

Schizo-affective Disorder	3

**Employed**	

Yes	4

No	37

**Pharmacological Treatment**	

Yes	40

No	1

**Receives Disability Pension**	

Yes	17

No	24

The criterion for inclusion in the study was being the main caregiver of the patient, i.e. the person who spends the most time supporting and taking care of the patient. Caregivers were excluded if they had an organic, sensory or severe cognitive disorder.

### Instruments

Assessments were conducted using the following instruments:

- *Zarit Burden Interview *(ZBI) [64], adapted and translated into Spanish and validated in Chile [65]. This instrument was used for its psychometric properties and the clarity of its statements. It has shown a high content and construct validity in different linguistic adaptations. In criterion validity studies, the ZBI has shown a high correlation with similar instruments (r = 0.71 with overall burden rate, r = 0.41 with Brief Symptom Inventory) were also closely related to detection of other disorders, mainly in mental health. An estimated 20.7% of mental disorders in caregivers, Zarit scale also can discriminate psychological distress with a sensibility of 93% and specificity of 80%. Although it was originally designed for caregivers of patients with dementia; the ZBI is now widely used to assess burden in relation to other disorders because of it ability to characterize the socio-cultural dynamics of the population to which it is applied. This makes it useful for the development of interventions. The ZBI comprises 22 items that explore the negative effects on caregivers in different areas of their life (physical, mental, social and economic). It comprises three subscales: Burden, which refers to the subjective impact of caring on the caregiver's life; Rejection, which includes items related to feelings of rejection/hostility towards the patient; and Competence, which is related to caregivers' self-assessment about their ability to maintain the relationship of care. Each statement is scored on a five-point Likert scale ranging from *never *to *almost always*. The total score is calculated by summing the responses to all items (score ranges from 22 to 110). The scale has satisfactory internal consistency, with a Cronbach's alpha of 0.91 for the global Burden and for the subscales the Cronbach's alpha: Burden = 0.90; Rejection = 0.71 and Competence = 0.69).

- *Attitudes Towards Schizophrenia Questionnaire for Relatives *(Additional File 1: Appendix 1). This instrument comprises 9 items rated on a Likert scale ranging from *strongly disagree *(1) to *strongly agree *(5). The final score is estimated by averaging the responses to all items (range 1-5). The items are mostly drawn from the *Family Attitude Scale (FAS) *[66], the *Questionnaire of Family Opinions (QFO) *[67], and the *Family Coping Questionnaire (FCQ) *[68]. Additional File 2: Appendix 2 shows the sources for the questionnaire items, as well as the attitude component to which each item belongs. In developing the questionnaire we decided to combine items from the abovementioned instruments and also added some new statements in order to obtain a questionnaire that considered the three attitude components. Given that most attitude questionnaires do not include items that assess behavioural features, we generated some of these and extracted others from the FCQ. Some modifications were also made due to the nature of the sample, since some of the behaviours referred to in the above questionnaires are not applicable to Chilean culture.

In short, this instrument aims to measure the attitudes of family members towards schizophrenia, considering the three attitude components: cognitive, behavioural and affective.

### Statistical Methods

In this study was used SPSS version 15 and AMOS version 5. First, we proceeded by analyzing the psychometric properties of the attitudes instrument with exploratory factor analysis, initially, allowing the reduction of the scale only 9 items, three sub-dimension, later performed by confirmatory factor and structural equation reliability, using Cronbach's alpha. Once the scale is tuned proceeded to t Studens with global attitude, taking as independent variable, the dichotomized variables (kinship and educational level) and correlation with numerical variables (age and years of living with the patient). The procedure was repeated with the components of the attitude scale, but instead of t student applies simple ANOVA. Also a multiple regression was performed with the enter method taking the global burden as the dependent variable and each of the dimensions of the attitude scale as independent variable.

### Procedure

The study was approved by the Ethics Committee of the University of Tarapacá and by each participating mental health service clinic.

Relatives of patients with schizophrenia were assessed while attending the mental health services in the city of Arica. Caregivers were introduced to the researcher by the head nurse of the psychiatric unit, who monitors the patients each month. The first author explained the purpose of the evaluation and invited relatives to participate. Informed consent was then obtained and it was made clear that all data would remain confidential. Caregivers were individually assessed with the ZBI and the *Attitudes Towards Schizophrenia Questionnaire for Relatives*. The researchers read the questions to the subjects because most of them had only a basic level of education. Each interview lasted assessment about 25 minutes.

## Results

The *Attitudes Towards Schizophrenia Questionnaire for Relatives *has a total score ranging from 1 a 5 and the lower the score, the better the attitude of the caregiver towards the patient and his/her disorder.

In order to assess the instrument's reliability we calculated Cronbach's alpha as an index of internal consistency. This gave α = 0.868 a value which did not improve when items were eliminated. Three subscales were formed based on the evident item content. In order to verify the validity of the subscale, Cronbach's alpha was calculated for each one:

• Behavioural component: α = 0.897

• Cognitive component: α = 0.903

• Affective component: α = 0.798

**Figure 1:**
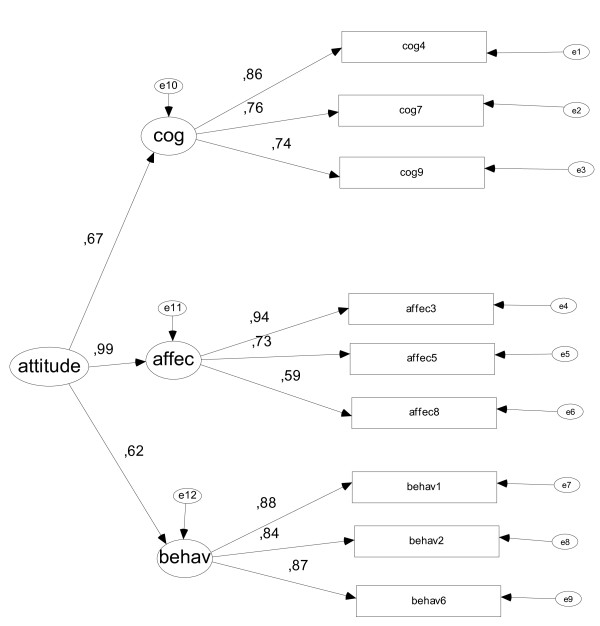
**Figure Construct validity Attitude Towards Schizophrenia Questionnaire for Relatives**. Figure Construct validity Attitude Towards Schizophrenia Questionnaire for Relatives (behav = behavioral; Affec = affective; cog = cognitive).

We also examined the construct validity using structural equation modeling using the AMOS program and method Estimation Scale-free least squares, being less demanding on the properties of multinormal (Figure 1). These analyses showed that the proposed structure had adequate fit to the data: GFI = 0.962, AGFI = 0.928, NFI (Delta1) = 0.940 and RFI (Rho1) = 0.910.

The analysis of attitude scores revealed significant differences related to certain variables: kinship, caregiver age, caregiver's level of education, and years living with the patient. Parents had a mean attitude score of 3.26 (SD = 0.90), whereas other caregivers (i.e. sons and daughters, siblings, couples and monitors) had a mean score of 1.86 (SD = 0.96). These differences are significant (t = 4.04, p < 0.000), showing that parents have a more negative attitude towards the disorder.

Caregivers with a low level of education had a mean attitude score of 3.42 (SD = 0.69), while those with a medium-high level had a mean of 2.37 (SD = 1.21) (t = 3.27; p < 0.003). Thus, caregivers with a higher level of education have a better attitude towards the patient and his/her disorder.

Elderly caregivers also showed a more negative attitude towards the disorder (r = 0.546; p < 0.000). Similarly, more years of living with the patient was associated with worse attitudes towards the patient and his/her disorder (r = 0.377; p = 0.015).

The remaining variables analysed, i.e., gender, employment and marital status of caregivers, and, among patients, gender, age, diagnosis, educational level, employment, receipt of a disability pension and number of hospitalizations, showed no significant association with the attitude score of caregivers in this sample.

We also examined differences between the three attitude components as regards the personal characteristics of caregivers and patients. The results revealed significant differences between the three components in relation to caregiver age, with older caregivers showing the most negative attitude. There were also significant differences related to the number of years the caregiver had lived with the patient: the longer they had lived with the patient the worse the caregivers' attitude was on cognitive and affective components. Parents showed a significantly worse attitude than did other relatives. Finally, caregivers with a low level of education showed a worse attitude towards the disorder. These data are shown in Table 3. The remaining variables, that is, gender, employment and marital status of caregivers, and, among patients, gender, receipt of a disability pension, the number of hospitalizations in recent years, educational level and age did not increase the differences between the three components.

**Table 3:** Caregiver and patient variables that produce differences in the components of the Attitudes Towards Schizophrenia Questionnaire for Relatives

	Behavioural component					Cognitive component					Affective component				
**Caregiver age**	r = 0.32*					r = 0.44**					r = 0.57**				

**Years living with the patient**	**r = 0.20 ns**.					r = 0.37*					r = 0.33*				

**Relationship**	**Parents**		**Others**		F = 4.66 p < 0.018	**Parents**		**Others**		F = 8.36 p < 0.002	**Parents**		**Others**		F = 19.75 p < 0.000
	*M*	*SD*	*M*	*SD*		*M*	*SD*	*M*	*SD*		*M*	*SD*	*M*	*SD*	
	3.07	1.42	1.92	1.32		3.09	1.30	1.78	0.68		3.63	0.95	1.88	1.31	

**Educational level**	**Low**		**Medium-high**		F = 8.41 p = 0.003	**Low**		**Medium-high**		F = 3.41 p < 0.036	**Low**		**Medium-high**		F = 10.93 p < 0.001
	*M*	*SD*	*M*	*SD*		*M*	*SD*	*M*	*SD*		*M*	*SD*	*M*	*SD*	
	3.36	1.33	2.12	1.36		3.13	1.29	2.38	1.24		3.76	0.67	2.59	1.52	

*P < .05; **p < .001 ns. = no signification

Finally, multiple regression analyses were conducted to assess the relationship between caregiver attitudes and burden. The model comprising the three components of the attitude questionnaire had significant but little explanatory power with respect to global burden, explaining 10% of the variance (R^2 ^= .104, *F *= 4,55, *p *= 0.039). These values are obtained by the enter method, leaving only a significant predictor, the affective component.

Regression analyses supported the correlation analyses, showing that the *affective *component was the best predictor of global attitude. Nevertheless, the predictive power of the model is limited, and it is likely that the abovementioned contextual variables are modulating the effect of attitudes. This hypothesis should be tested in future research with a larger sample.

## Discussion

Community care for patients with schizophrenia relies heavily on the informal care provided by relatives, which supplements the shortage of medical, occupational and residential resources. Indeed, the idea that patients remain with their families has become an end in itself, regardless of its viability [36,54].

However, the closure of mental hospitals and the assumption of the role of caregivers by families has revealed the phenomenon of caregiver burden [60]. Since the early 1950s many studies have provided evidence about the negative impact of caregiving on the families of patients with schizophrenia, especially among those who assume the role of main caregiver [60,69]. Research has also examined the relationship between burden and caregivers' demographics and patients' symptoms [40,70-72]. In this regard, some recent studies have suggested that a minor role should be attributed to the socio-demographic and psychosocial factors that influence burden.

At all events, there is renewed interest in the role played by the family in the community management of mental disorders, and specifically in the way that families deal with caregiving [13,39,40,73].

The present study of caregiver's attitudes, showed that these attitudes tend to be negative in the sample as a whole. When considering the three components of attitudes the most negative caregiver attitudes were associated with the affective component. This could act as a trigger for depressive symptoms, suicidal ideation and substance abuse in relatives [74], especially in those caregivers with low levels of education [75].

In addition, analysing the characteristics of caregivers and/or patients that might modulate these attitudes, the results show that mothers were the caregivers with the most negative attitude towards schizophrenia. This is because a large percentage of them are primary caregivers, taking responsibility for all the care of their schizophrenic child. This is related to the greater involvement of mothers, both psychologically and in practice (in their role) [76], and is an aspect that needs to be addressed more actively by mental health professionals [38,77-79]. Some authors have even argued that the care given by mothers is a 'moral obligation' within a patriarchal society, in that this care towards others represents the way they connect with the social sphere [80]. Whatever the case, most studies report that the mother is the one who takes care of the patient [81-85], and even when they share the patient's care with other relatives it is likely that the woman, the primary caregiver, experiences a greater burden and, therefore, a more negative attitude.

Attitudes were also associated with caregiver's age and the number of years that the caregiver has lived with the patient. Specifically, older caregiver who had spent more time with the patient had more negative attitudes towards the disorder. Thus, the extended exposure of caregivers in this study resulted in a negative attitude towards both the patient and schizophrenia.

The results also showed that a higher level of education among relatives was an indicator of a better attitude towards the disorder. This can be explained by the fact that caregivers with more education have access to a wider range of information about the disorder and/or to community resources which they could turn to for help. These results are consistent with the findings of Magliano and colleagues [68], who observed a more positive attitude towards patients among relatives with higher levels of education. By contrast, low levels of education and lower age predict depressive symptoms, leading to high levels of burden and negative attitudes [75].

## Conclusions

Given these findings, one is obliged to ask: Who takes care of the caregiver? Furthermore, if caregivers suffer an emotional breakdown or are at risk of developing psychopathology, how can they play their role properly? The present research shows that perceived burden in the caregiving role is significantly correlated with global attitude. The worse the attitude towards the patient, the more concerned the caregiver is about his/her competence in the role. It is here, therefore, that psycho-educational programmes can make a valuable contribution. By giving them the tools they need to manage the disorder, it would be possible to improve caregivers' attitudes, reduce their burden and, eventually, enable a better relationship between patient and caregiver.

Despite the interest of the results obtained, several limitations of the study should be noted. First, the sample is small and may not be representative of the general population, but rather of a group of people with specific socio-cultural characteristics. Second, the main results are based on scores obtained on the *Attitudes Towards Schizophrenia Questionnaire for Relatives *and its three components. This questionnaire was developed with the purpose of assessing attitudes related to cognitive, behavioural and affective aspects. However, the small sample size undermines the validation process followed when developing the questionnaire. In light of these limitations the results obtained must be interpreted with caution. Future research would need to consider the mediating role of contextual variables such as kinship, age, years of living with the patient, and educational level.

Although psycho-educational programmes have proved to be effective they have yet to be widely implemented in Chile as a formal part of the country's mental health policy, this being especially the case in some northern regions. Moreover, a scarcity of resources has meant that the focus is exclusively on the patient's symptoms, and therefore an integrated approach to treatment remains a remote reality in this socio-cultural context, particularly in the regions. Most of the initiatives that have been implemented with caregivers have been linked to research projects in which the government has made a significant investment. However, there is now a need for the strategic intervention plans developed by policy makers to be based on empirical evidence, thereby enabling social and mental health services to promote better equity, quality and efficiency [3]. It is in this sense that the present study is intended to be a first step toward providing further evidence of the need for psychological intervention with caregivers of patients with schizophrenia.

## Abbreviations

QOL: Quality of Life; EE: Expressed Emotion.

## Competing interests

The authors declare that they have no competing interests.

## Authors' contributions

ACU contributed to the design and coordination of the study. JGM was responsible for the primary study design and supervision of data collection. MFG acted as a methodology consultant and assisted with data analysis and interpretation. CPS participated in manuscript editing. DRA conducted and tabulated the literature search. ACP participated on statistical analysis and interpretation. All authors read and approved the final manuscript.

## Pre-publication history

The pre-publication history for this paper can be accessed here:

http://www.biomedcentral.com/1471-2296/12/101/prepub

## Supplementary Material

Additional file 1 **Attitudes towards Schizophrenia Questionnaire for Relatives**. Appendix 1 shows the instrument that comprises 9 items rated on a Likert scale ranging from *strongly disagree *(1) to *strongly agree *(5). The final score is estimated by averaging the responses to all items (range 1-5). Click here for file

Additional file 2 **Questionnaires of origin and their respective components**. Appendix 2 shows the sources for the questionnaire items, as well as the attitude component to which each item belongs. Click here for file
