# Supplementary material for: Attitudes and burden in relatives of patients with schizophrenia in a middle income country
Source: BMC Fam Pract. 2011 Sep 26;12:101. doi: 10.1186/1471-2296-12-101 (PMC3191329; doi:10.1186/1471-2296-12-101)
Supplement: Additional file 1 — Attitudes towards Schizophrenia Questionnaire for Relatives. Appendix 1 shows the instrument that comprises 9 items rated on a Likert scale ranging from strongly disagree (1) to strongly agree (5). The final score is estimated by averaging the responses to all items (range 1-5). [file 1471-2296-12-101-S1.DOCX]

**Appendix 1**

**Title:** Attitudes towards Schizophrenia Questionnaire for Relatives

**Description:** Appendix 1 shows the instrument that comprises 9 items rated on a Likert scale ranging from *strongly disagree* (1) to *strongly agree* (5). The final score is estimated by averaging the responses to all items (range 1-5).

Below are a series of statements. Please choose and circle the answer that best describes your opinion. Try to answer as honestly as possible and remember that the information is strictly confidential.

**Example:**

**Work should be well paid**

1 2 3 4 5

I strongly disagree I disagree I don’t know I agree I strongly agree

**All TV programmes are good**

1 2 3 4 5

I strongly disagree I disagree I don’t know I agree I strongly agree

**1. I avoid engaging in conversation with my disturbed relative**

1 2 3 4 5

I strongly disagree I disagree I don’t know I agree I strongly agree

**2. When he/she becomes rude, I am aggressive with him/her**

1 2 3 4 5

I strongly disagree I disagree I don’t know I agree I strongly agree

**3. I am tired of trying to control him/her all the time**

1 2 3 4 5

I strongly disagree I disagree I don’t know I agree I strongly agree

**4. Patients with schizophrenia should always be hospitalized**

1 2 3 4 5

I strongly disagree I disagree I don’t know I agree I strongly agree

**5. I prefer to hide the illness from the rest of my family and friends**

1 2 3 4 5

I strongly disagree I disagree I don’t know I agree I strongly agree

**6. I really try to enjoy being with him/her at home**

1 2 3 4 5

I strongly disagree I disagree I don’t know I agree I strongly agree

**7. My relative causes problems deliberately**

1 2 3 4 5

I strongly disagree I disagree I don’t know I agree I strongly agree

**8. I don’t like to talk with anybody about the things that happen to my relative**

1 2 3 4 5

I strongly disagree I disagree I don’t know I agree I strongly agree

**9. My relative’s disturbance will worsen over time**

1 2 3 4 5

I strongly disagree I disagree I don’t know I agree I strongly agree

**Scoring key:** The scores for each item range from 1 to 5, where 1 is the positive pole and 5 is the negative 1. The total score is the mean of all items.
