# Supplementary material for: Attitudes and burden in relatives of patients with schizophrenia in a middle income country
Source: BMC Fam Pract. 2011 Sep 26;12:101. doi: 10.1186/1471-2296-12-101 (PMC3191329; doi:10.1186/1471-2296-12-101)
Supplement: Additional file 2 — Questionnaires of origin and their respective components. Appendix 2 shows the sources for the questionnaire items, as well as the attitude component to which each item belongs. [file 1471-2296-12-101-S2.DOCX]

**Appendix 2**

**Title:** Instruments from which we took the items for the Attitudes Towards Schizophrenia Questionnaire for Relatives, and the attitude component to which they belong

**Description:** Appendix 2 shows the sources for the questionnaire items, as well as the attitude component to which each item belongs.

| **Item** | **Questionnaire of origin** | **Component of origin** |
| --- | --- | --- |
| **1** | Family Coping Questionnaire [56] | Behavioural |
| 2 | New | Behavioural |
| **3** | New | Affective |
| **4** | Questionnaire of Family Opinions [55] | Cognitive |
| **5** | New | Affective |
| **6** | Family Attitude Scale [54] | Behavioural |
| **7** | Family Attitude Scale [54] | Cognitive |
| **8** | New | Affective |
| **9** | Family Coping Questionnaire [56] | Cognitive |
